# High-accuracy source-independent radiometric calibration with low complexity for infrared photonic sensors

**DOI:** 10.1038/s41377-021-00597-4

**Published:** 2021-08-09

**Authors:** Qiang Guo, Fuchun Chen, Xiangyang Li, Boyang Chen, Xin Wang, Guilin Chen, Caiying Wei

**Affiliations:** 1 National Satellite Meteorological Center, China Meteorological Administration, 100081 Beijing, China; 2 Key Laboratory of Infrared System Detection & Imaging Technology, Shanghai Institute of Technical Physics, Chinese Academy of Sciences, 200083 Shanghai, China; 3 Key Laboratory of Infrared Imaging Materials & Detectors, Shanghai Institute of Technical Physics, Chinese Academy of Sciences, 200083 Shanghai, China

**Keywords:** Imaging and sensing, Optical sensors

## Abstract

Radiometric calibration (RC) is an essential solution to guarantee measurements from infrared photonic sensors with certain accuracy, the main task of which is to determine the radiometric responsivity of sensor and usually be solved by comparing with some radiation source (i.e., blackbody), called source-based RC (SBRC). In addition to the complexity in manufacture, the nonideal characteristics of an available source will inevitably introduce unexpected uncertainties to reduce the final calibration accuracy by around 0.2–0.5 K in SBRC. Therefore, we propose an original source-independent RC (SIRC) principle based on modeling instead of comparing for SBRC, where the incident background radiation to detector, as a dominated factor influencing the responsivity characteristics of a photonic sensor, is modeled to implement RC for both two fundamental types (photoconductive and photovoltaic) of HgCdTe photonic detectors. The SIRC merely requires the temperature information of main components of a sensor other than some complex source and its assembly, and provides a traceable way at lower uncertainty costs relative to the traditional SBRC. The SIRC is being implemented in Fengyun-2 satellites since 2019, which ensures a long-term stable service of Chinese geostationary meteorological satellites for the global observation system under the framework of World Meteorological Organization. Moreover, a 20-year-period traceable Fengyun-2 dataset to be recalibrated with SIRC will benefit the further climate applications.

## Abstract (web summary)

A high-accuracy source-independent radiometric calibration (SIRC) methodology with low complexity for both photoconductive and photovoltaic infrared photonic sensors is proposed to provide an easier SI-traceable way for global observation systems.

## Graphical abstract

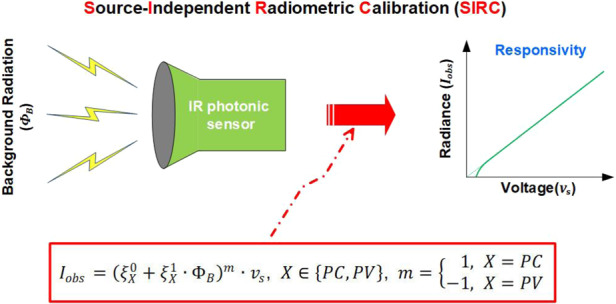

## Introduction

Infrared (IR, particularly referring to the spectral range between 3 and 15 microns) measurements from different in-orbit sensors are generally regarded as one of the most important spaceborne remotely sensed information for monitoring meteorological and environmental disasters, investigating surface parameters (i.e., emissivity and temperature) of some targeted scenes^1^ as well as determining the temperature and humidity structures of atmosphere^2,3^ with hyperspectral soundings within IR band^4^. Moreover, during the last decade, severe demands on IR measurements of sufficient accuracy and sensitivity to allow reliable detections of climate changing (i.e., the time scale, consequences, and causal attribution) can be ensured by the International System of Units (SI) traceability of sensors onboard some new satellite missions, i.e., infrared spectrometer on CLARREO (Climate Absolute Radiance and Refractivity Observatory) spacecraft^5^. In order to achieve such above goals as close as possible, sensor-specific calibration is therefore required to provide accurate enough or even SI-traceable quantities related to the acquired measurements for end users. As defined by the Working Group on Calibration and Validation (WGCV) of the Committee on Earth Observation Satellites (CEOS), calibration can be described as the process of quantitatively defining the system response to known, controlled signal inputs^6^, which at least includes radiometric, spectral, and spatial response aspects^7^. Particularly, as for the radiometric response calibration (or simply called radiometric calibration, RC), it is composed of four primary components, i.e., preflight RC, in-flight RC, vicarious RC, and intercalibration, specifically:

The preflight RC is implemented at the facilities of payload vendor prior to launch^8^, the aims of which are to test or validate its main specifications, i.e., sensitivity, linearity, spectral response function, and modulation transfer function^9^ as well as to determine the response relationship between instrument output and radiant input^10^, especially the quadratic nonlinearity term, which is hardly obtained under in-orbit condition for most sensors and usually can be deduced here by viewing a well-characterized radiant source at a series of temperature situations in a relatively large range^11^.

Since the radiometric response relationships from preflight RC in IR band may change for an in-orbit IR sensor due to the variations in its environment surroundings^12^, it is therefore necessary to perform in-flight RC, and most spectral imagers on operational meteorological satellites use onboard blackbody (BB) references to monitor and adjust the RC coefficients for IR bands on a frequent basis (at the order of 10^0^–10^1^ m)^13^. Specifically, for the first-generation spin-stabilized meteorological satellites (i.e., GOES-4/−5^14,15^, MFG^16^, GMS-5^17,18^, and FY-2^19^), the onboard IR wide-band radiometers are equipped with internal or so-called partial-path BBs as references for implementing RC, the accuracies of which can approach around 1–3 K. Thereafter, the improved full-path BB scheme is successively adopted in imagers onboard the second-generation three-axis stabilized ones, including GOES-8/−9^20^, Hamawari-8^21^, and FY-4A^22^, and their RC accuracies for their main IR bands behave well, lower than 0.5 K in general. For other IR sensors equipped on some environmental-related satellite missions, i.e., ATSR^23^ and its improved one (AATSR^24^on ERSs, ETM+^25^on Landsat, VISR^26^ on TRMM and MODIS^27^ on Terra and Aqua, some perfect-qualified but nonideal BBs are utilized for in-flight calibration, while the calibration accuracies in IR bands of VIRS^28^ and MODIS^29,30^ are evaluated within some relatively long-term periods. Besides such a normal BB source for in-flight IR calibration in diverse missions, some standard stars (i.e., Vega and Sirius) are proposed for IR calibration of some space-based sensors^31^, which are not adoptable for most Earth observation missions.

Vicarious calibration is performed with some natural or artificial sites on the surface of the Earth usually at the stage of post launch, the two main solutions of which are temperature- and radiance-based methods^32^. Due to inevitable usage of other sources (i.e., a radiative transfer model, atmospheric profiles measured by a radiosonde and nonstrict viewing geometry from an aircraft) in IR vicarious calibration, its accuracy is always around 2–3 K which of course limits its applicable situations^33^.

Intercalibration between different sensors in space aims to make all the measurements SI-traceable or at least with an identical radiometric standard^34^, where unifying the spectral responses between the monitored and the reference instruments can be implemented with two different ways, i.e., the radiative transfer modeling method^35^ and the high spectral convolution (HSC) one recommended by the Global Space-based Inter-Calibration System (GSICS)^36^. With a certain improvement in spatial collocation^37^, the HSC method has been universally applicable for most meteorological satellites to increase the confidence in their operational calibrations^38^, and to help modify their possible spectral shifts in different IR bands due to a certain unexpected contaminations from deep space situations^39,40^.

In fact, according to the definition of calibration given by WGCV, the calibrated “system response” is the sole feature of the sensor itself, while the “known and controlled input signal” merely acts as a reference to provide a feasible way for implementing calibration. Therefore, in essence, the characteristics of the sensor’s response are independent of such a known and controlled source, which implies that we may obtain them in other ways, e.g., modeling with the dominant impact factors, instead of the traditional one by using measurement with a source (called source-based RC, SBRC). Although the source-independent RC (SIRC) is theoretically feasible, few applicable cases in such a method have been reported so far, particularly for operational space-to-Earth sensors. However, in June 2018, the employed source (i.e., internal blackbody) for in-orbit RC of the G satellite of the Fengyun-2 series (FY-2G) was invalid and no longer for reference due to some unexpected fatal causes (Supplementary Note 1). In fact, such a situation would lead to no calibrated radiance of the four infrared bands from its main payload (i.e., Visible Infrared Spin-Scan Radiometer, VISSR) for end users unless a substitute calibration method with comparable accuracy could be adopted. Driven by such an emergent requirement, we propose an original SIRC for the IR bands of FY-2G satellite for utilization since January 2019, the background-limited capabilities of which are exploited to establish a new model describing the radiometric response of each individual band with a certain relevant parameters. Thereafter, in January 2020, the proposed SIRC method was further adopted in the F satellite of the Fengyun-2 series (FY-2F) for operational uses to enhance its calibration accuracies in IR bands as well as to increase its calibration frequency especially under the condition of regional scanning mode (Supplementary Note 2). Fortunately, the operational implementation of SIRC guarantees a long-term stable application of Chinese geostationary meteorological satellites for the global observation system under the framework of World Meteorological Organization, and further supports the meteorological services from Fengyun-2 (FY-2) satellites for “B&R” countries. Moreover, the SIRC method is expected to recalibrate the measurements from all the FY-2 satellites to establish a 20-year-period traceable dataset for climate application.

This article aims to provide a novel SIRC method for an IR photonic sensor with a closely background-limited capability, which is easy to be achieved by most similar instruments in space thanks to their low enough working temperatures less than 90 K in general. Relative to the traditional SBRC with a full- or partial-path BB, the high accuracy of the proposed SIRC is ensured by its possible uncertainty contributors merely from temperature measurement, while the additional ones introduced by source (i.e., BB) itself will be eliminated. Meanwhile, the low complexity of SIRC is accomplished that the ordinary calibration BB source and its assembly are no longer required.

## Results

### Methodology for SIRC of background-limited IR measurements

All objects with their temperatures higher than absolute zero are continually emitting infrared radiation, which is microscopically composed of a large number of photons with a wavelength distribution. The interaction of infrared radiation with electrons results in different photoeffects, i.e., photoconductive (PC), photovoltaic (PV), and so on. Based on these photoeffects, however, only PC and PV detectors have been widely exploited^41^. In essence, the PC detector and the PV one is a radiation-sensitive resistor and diode, respectively. Specifically, when a photon (its energy greater than the bandgap energy of semiconductor is required for an intrinsic PC detector) is absorbed to produce electron-hole pairs, the electrical conductivity of PC detector and the voltage across the p-n junction of PV detector are correspondingly changed^42^. During the last several decades, the popularity of HgCdTe (a direct bandgap ternary-alloy semiconductor material, mercury cadmium telluride, MCT) detectors is made possible by their flexibility in spectral response over a wide span of the infrared regions of interest. Three generations of HgCdTe devices have been successively developed, where the first ones consist of a single, multiple, or linear arrays of PC/PV device(s), the second ones are two-dimensional arrays of PV detectors, and the third ones are not strictly defined but have substantially enhanced capabilities over the second ones^43^. Until recently, for most IR sensors on a variety of earth satellite missions (i.e., VISSR^19^and MODIS^27^, more information provided in Supplementary Note 3), only the first-generation of HgCdTe PC and PV detectors are operationally utilized for the purposes of the high accurate IR imaging or sounding measurements, where the severe nonuniformity in spectral response among huge number of detectors in a sensor array may be one primary defect to limit the comprehensive applications of the second- and third-generations of HgCdTe detectors in space uses.

In usual, for a spaceborne IR sensor, the deployed HgCdTe PC/PV detectors are required to be performance-limiting components, which means it is necessary to use detectors with a sensitivity limited merely by the random rate of arrival of photons from the scene, known as the background-limited infrared photodetector (BLIP) limit. In order to achieve such an ultimate performance, the thermal and amplifier noises of a detector should be low compared to the photon noise, which arises from the detection process itself, as a result of the discrete nature of the radiation field. Meanwhile, detectors with or at least close to the BLIP performance are recommended to operate at a temperature in the range 80–140 K^42^, which is currently easy to be satisfied for most onboard IR sensors.

**Fig. 1:**
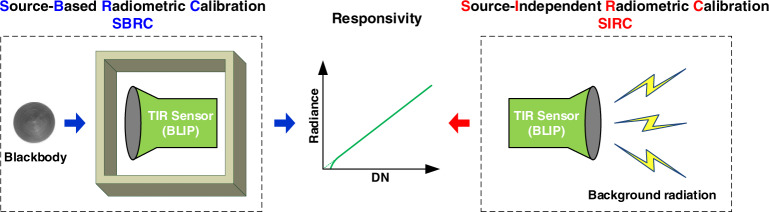
Comparison of working mechanisms between SBRC and SIRC for a BLIP sensor. In SBRC, the responsivity of an IR sensor is measured as a black box to obtain its whole output when viewing the radiation from a source (i.e., blackbody). Conversely, in SIRC, the responsivity of a full or at least closely approached BLIP sensor, which can be satisfied by most onboard sensors, is calculated by modeling the relationship between the incident background radiation and its responsivity. In essence, SBRC is a measurement-based method while SIRC is a modeling-based one. Particularly, the responsivity here merely refers to the linear component of the whole relationship between DN and radiance illustrated above

Based on the operating principles of a background-limited sensor in IR band (or called BLIP sensor), its core performances (i.e., detectivity and responsivity) are absolutely dominated by the surrounding radiation field viewable by each detector. Therefore, relative to the traditional SBRC with a known radiation source, we propose an original SIRC method for a BLIP sensor where the responsivity of the individual detector can be described and further determined by incident background radiation, instead of using a referenced radiation source. Figure 1 illustrates the working mechanism comparison between SBRC and SIRC methods. Specifically, on one hand, under the framework of SBRC, such an IR senor is regarded as a black box, the performances (such as the responsivity for RC) of which are measured by comparing with a referenced source (i.e., blackbody). It should be emphasized that the SBRC is universally suitable for all IR sensors (BLIP or not), although most of them have the BLIP capabilities. On the other hand, in the SIRC method, the RC responsivity of an IR sensor can be determined by modeling with multiple impact factors including its background radiation, which is uniquely applicable for a full or at least closely approached BLIP sensor in theory. Apparently, the “comparing” mode of SBRC is replaced by the “modeling” one of SIRC while the ordinary radiation source and its related assemblies are no longer in need to simplify the design as well as fabrication of a sensor significantly. Moreover, due to the inherent characteristics in aspects of both the nonlinearity and the dark current effects for a realistic infrared photonic sensor, the relationship between digital number (DN) and radiance in Fig. 1 is not always constant, particularly when DN is relatively small or at least approaches zero. Such an inconstant responsivity is beyond the capabilities of SBRC as well as SIRC methods, and usually can be corrected by using the prelaunch measurements.

According to the basic definitions for photodetector figures of merit^41,42^, the relationship between the output voltage (*v*_*s*_) for PC detector or current (*i*_*s*_) for PV detector and an observed target whose spectral radiance can be given by *I*_*obs*_ with unit of ${{{\mathrm{W}}}} \cdot {{{\mathrm{m}}}}^{ - 2} \cdot {{{\mathrm{sr}}}}^{ - 1}$ is provided in Supplementary Note 4. For a PC-type BLIP sensor, its total output noise level (*v*_*n*_), which usually consists of three main components for a PC detector, i.e., the thermal noise, the 1/*f* noise, and the generation and recombination noise $\left( {v_{g - r}} \right)$, is dominated by $v_{g - r}$^44^. Based on the theoretical derivations provided in Supplementary Note 5, we can draw

1 $$I_{{\mathrm{obs}}} = \left( {\xi _{PC}^0 + \xi _{PC}^1 \cdot {{{\mathrm{{\Phi}}} }}_B} \right) \cdot v_s$$

In Eq. (1), ${{{\mathrm{{\Phi}}} }}_B$ is the background photon flux, $\xi _{PC}^0$ and $\xi _{PC}^1$ are reasonably regarded as the positive constants to be determined.

Furthermore, according to the operating mechanism of a PV-type sensor, its quantum efficiency is definitely variable with its background radiation while that of PC-type sensor is usually constant, and is contributed by three regions, i.e., two neutral regions of different types (p and n) of conductivity and the spatial charge region. Based on the theoretical derivations provided in Supplementary Note 6, we can also draw

2 $$I_{{\mathrm{obs}}} = \left( {\xi _{PV}^0 + \xi _{PV}^1 \cdot {{{\mathrm{{\Phi}}} }}_B} \right)^{ - 1} \cdot v_s$$

In Eq. (2), the parameters ${\upxi}_{{{{\mathrm{PV}}}}}^0$ and ${\upxi}_{{{{\mathrm{PV}}}}}^1$ are doubtlessly regarded as the constants to be determined, where the former is positive and the latter is negative.

By using Eqs. (1) and (2), a uniform radiometric response formula for both PC- and PV-types BLIP system can be given by

3 $$\begin{array}{ll}I_{{\mathrm{obs}}} = \left( {\xi _X^0 + \xi _X^1 \cdot {\Phi} _B} \right)^m \cdot v_s, \in \left\{ {PC,PV} \right\},m = \left\{ {\begin{array}{l} {1,X = PC} \\ { - 1,X = PV} \end{array}} \right.\end{array}$$

As for the ${{{\mathrm{{\Phi}}} }}_B$ parameter in Eq. (3), it consists of all the viewable radiation from different surrounding components upon the photon detector itself with the unit of photon number and can be given by

4–1 $${{{\mathrm{{\Phi}}} }}_B = \mathop {\sum}\limits_i {{{{\mathrm{{\Phi}}} }}_B^i} = \mathop {\sum}\limits_i {\left[ {\zeta _i \cdot \mathop {\int}\nolimits_{\lambda _i^1}^{\lambda _i^2} {srf_i\left( \lambda \right) \cdot Q\left( {\lambda ,T_i} \right) \cdot d\lambda } } \right]}$$

4–2 $$Q\left( {\lambda ,T} \right) = \frac{{2\pi c}}{{\lambda ^4}} \cdot \frac{1}{{e^{hc/\lambda kT} - 1}}$$

In Eq. (4–1), *srf*_*i*_ and *T*_*i*_ are the spectral response function and equivalent temperature of *i*th surrounding component viewable by the detector, $\lambda _i^1$ and $\lambda _i^2$ are the wavelength ranges of $srf_i$, $\zeta _i$ is the radiant contribution coefficient for individual viewable component, which is mainly dominated by the optical path attenuation and the subtended solid angle upon the detector. In Eq. (4-2), Q(*λ*,*T*) is the emittance of a radiation source given by photon number, *h* is the Planck constant, *c* is the velocity of light and *k* is the Boltzmann constant.

In fact, for a well-designed BLIP spaceborne sensor, its radiometric calibration formula is usually satisfied with linear relationship between the observed radiation (*I*_*obs*_) and the output voltage (ν_*s*_) at least within the specific detecting range, i.e.,

5 $$I_{{\mathrm{obs}}} = CAL_{{\mathrm{slope}}} \cdot v_s - CAL_{{\mathrm{offset}}}$$

where *CAL*_*slope*_ is the main calibration parameter to be determined firstly, and *CAL*_*offset*_ almost approaches zero for the most cases and can be estimated by multiplying the root mean square of the total noise of sensor in voltage by *CAL*_*slope*_^19^, where the former is usually calculated when viewing a known target (i.e., cold space). Using Eqs. (3–5), we can draw

6 $$\begin{array}{ll}{{CAL}_{\mathrm{slope}}} \,=\, \left( {\xi _X^0 + \xi _X^1 \cdot {{{\mathrm{{\Phi}}} }}_B} \right)^m \\ \qquad\qquad\,\,=\, \left\{ {\xi _X^0 + \mathop {\sum}\limits_i {\left[ {\left( {\xi _X^1} \right)_i \cdot \mathop {\int}\nolimits_{\lambda _i^1}^{\lambda _i^2} srf_i\left( \lambda \right) \cdot Q\left( {\lambda ,T_i} \right) \cdot d\lambda } \right]} } \right\}^m,i \in \left[ {1,N_c} \right]\end{array}$$

where $\left( {\xi _X^1} \right)_i$ is constant to be determined for *i*th surrounding component $\left\{ {\left( {\xi _X^1} \right)_i = \xi _X^1 \cdot \zeta _i} \right\}$ of the total *N*_*c*_ ones. Nevertheless, a novel SIRC method (mainly for the calibration slope) for a BLIP sensor is originally established where the surrounding temperature fields are utilized, independent of a blackbody source which is universally adopted as a radiometric reference in the traditional SBRC one.

### Determination of main parameters for SIRC

In general, there are two fundamental steps for SIRC algorithm. The first one is to determine the main parameters given in Eq. (6) with some reliable reference measurements before or after launch. The second one is to utilize those determined parameters together with some necessary environmental temperature information to generate the calibration results, i.e., calibration slope and calibration offset. Specifically, although the constant parameters in Eq. (6), i.e., $\xi _X^0$ and $\{ {\left( {\xi _X^1} \right)_i} \}$ could be calculated according to their definitions, it is better to determine them with some available accurate well-calibrated results from either in-lab or in-orbit situation. Specifically, for a group of well-calibrated results (i.e.,$\left\{ {CAL_{{\mathrm{slope}}}^j,j \in \left[ {1,N_s} \right]} \right\}$) from the different environmental temperature fields, which means that the equivalent temperatures $\left\{ {T_i^j} \right\}$ of the individual viewable surrounding components diverse with each other, Eq. (6) can be rewritten as

7 $$\begin{array}{ll}\left\{ {\begin{array}{*{20}{c}} {\xi _X^0 + \mathop {\sum}\limits_{i \in \left[ {1,N_c} \right]} {\left[ {\left( {\xi _X^1} \right)_i \cdot \mathop {\int}\nolimits_{\lambda _i^1}^{\lambda _i^2} {srf_i\left( \lambda \right) \cdot Q\left( {\lambda ,T_i^1} \right) \cdot d\lambda } } \right]} = \left( {CAL_{{\mathrm{slope}}}^1} \right)^m} \\ \cdots \\ {\begin{array}{*{20}{c}} {\xi _X^0 + \mathop {\sum}\limits_{i \in \left[ {1,N_c} \right]} {\left[ {\left( {\xi _X^1} \right)_i \cdot \mathop {\int}\nolimits_{\lambda _i^1}^{\lambda _i^2} {srf_i\left( \lambda \right) \cdot Q\left( {\lambda ,T_i^j} \right) \cdot d\lambda } } \right]} = \left( {CAL_{{\mathrm{slope}}}^j} \right)^m} \\ \cdots \\ {\xi _X^0 + \mathop {\sum}\limits_{i \in \left[ {1,N_c} \right]} {\left[ {\left( {\xi _X^1} \right)_i \cdot \mathop {\int}\nolimits_{\lambda _i^1}^{\lambda _i^2} {srf_i\left( \lambda \right) \cdot Q\left( {\lambda ,T_i^{N_s}} \right) \cdot d\lambda } } \right]} = \left( {CAL_{{\mathrm{slope}}}^{N_s}} \right)^m} \end{array}} \end{array}} \right.\\ m = \left\{ {\begin{array}{*{20}{c}} {1,X = PC} \\ { - 1,X = PV} \end{array}} \right.\end{array}$$

Mathematically, to achieve at least one set of real solutions of $\xi _X^0$ and $\{ {\left( {\xi _X^1} \right)_i} \}$, *N*_*s*_ ≥ *N*_*c*_ + 1 should be satisfied. In practice, Eq. (7) can be solved with the generalized inverse method based on singular value decomposition technique for matrix^45^.

Here, we use FY-2, which is the first-generation geostationary meteorological satellite series developed in China, as an example to implement the proposed SIRC method to fulfill its in-orbit RC in IR bands. So far, eight members of this family have been launched since 1997, four of which, i.e., FY-2E/F/G/H are in operation or as backup on different orbital positions. VISSR is the main payload of FY-2 satellite and can acquire the radiation information of atmosphere, cloud, ocean, and land surfaces in visible and IR spectrums within the full-Earth-disc region. For the normal (space or Earth view) observation, the target’s radiation is gathered by VISSR in form of parallel beam and then converged to the detectors by three-time reflection by the primary mirror (PM), the second mirror (SM), and the folding mirror (FM) in order, as well as one penetration of the relay lens (RL). Supplementary Note 7 provides more information about the basic observation mechanism of FY-2 satellites, where the dominated background radiation is mainly contributed from both RL and SM of VISSR.

Due to the invalidation of the in-orbit internal BB function for FY-2G since June 2018 (can be referred in Supplementary Note 1) and lack of effective internal BB view for FY-2F during its frequent regional scanning observations, the SIRC method was therefore applied to FY-2G and FY-2F satellites in January 2019 and January 2020, respectively. By using Eq. (7), in order to achieve the necessary calibration coefficients of SIRC (i.e. $\{ {CAL_{{\mathrm{slope}}}^j,j \in \left[ {1,N_s} \right]} \}$, $\left( {\xi _{VISSR}^1} \right)_{RL}$ and $\left( {\xi _{VISSR}^1} \right)_{SM}$) for most bands (i.e., IR1: 10.3–11.3 μm, IR2: 11.5–12.5 μm, and IR3: 6.3–7.6 μm) of both FY-2G and FY-2F satellites, all the available corrected calibration results are determined within a complete 1-year period. Particularly, these results were originally generated by IBBC method^19^ and then corrected by intercalibrating with other reference sensors (called intercalibrated results)^37^. This is expected to make the deduced coefficients more reliable and suitable for all the possible situations. Specifically, the period from June 2017 to May 2018 is selected for FY-2G, the calibration coefficients of which are listed in Table 1. Apparently, all the coefficients are positive as expected for PC BLIP system, and $\left( {\xi _{VISSR}^1} \right)_{RL}$ is generally larger than $\left( {\xi _{VISSR}^1} \right)_{SM}$, which is quite reasonable thanks to the more background radiation contribution of a penetrative component (RL) than that of a reflective one (SM). For all the four infrared bands of FY-2G satellite, the typical values of emissivity of SM are around 0.02 measured before launch while those of RL approach 0.04, the relationships of which are generally identical to the calibration coefficients listed in Table 1.

**Table 1:** Main SIRC coefficients for IR1–IR3 bands of both FY-2G and FY-2F satellites with the intercalibrated results during different time periods

Satellite	Label	Time period	Band	$\xi _{VISSR}^0$	$\left( {\xi _{VISSR}^1} \right)_{RL}$	$\left( {\xi _{VISSR}^1} \right)_{SM}$
FY-2G	Unique	2017/06/01–2018/05/31	IR1	1.561097	2.984730	1.084689
			IR2	1.252979	3.636097	0.519565
			IR3	1.075739	1.420327	1.010236
FY-2F	No.1	2017/01/01–2017/12/31	IR1	2.068301	2.606485	0.328674
			IR2	1.768189	2.770642	0.092834
			IR3	1.122187	1.715914	0.470124
	No.2	2017/08/01–2018/07/31	IR1	1.974135	2.670247	0.506463
			IR2	1.743943	2.877651	0.084166
			IR3	1.113357	1.718011	0.522496
	No.3	2018/01/01–2018/12/31	IR1	2.054748	2.637165	0.346619
			IR2	1.837994	2.795410	0.046004
			IR3	1.004046	1.737685	1.057612

For FY-2F satellite, to evaluate the deduced coefficients from the intercalibrated results in different period of time, three sets of coefficients (labeled with No. 1, No. 2, and No. 3) from January to December 2017, August 2017 to July 2018, and January to December 2018 are calculated and listed separately in Table 1. In fact, since the intercalibrated results to generate the calibration coefficients are not exactly identical with each other among the three periods, a certain or a general small difference among the three sets are inevitable, except that the coefficients of IR3 band for No. 3 case differ in a relatively large manner from those of No. 1 and No. 2. To evaluate the impacts of different coefficients on SIRC, 12 typical cases at the beginning of every month in 2019 with different temperature fields are selected, and the corresponding calibration slopes of SIRC are provided in Table 2, where an indicator of relative variation for the three slopes (the mean value is divided by the standard deviation, namely *σ*/mean) is also calculated for different bands of each case. Specifically, the maximum value of *σ*/mean of the three bands is less than 5‰ and the mean ones are 0.93‰, 3.04‰, and 2.04‰ for IR1, IR2, and IR3 bands, respectively, which are equal to the BT biases less than 0.2 K for the three bands at different reference temperatures for each as shown in Table 2. Considering that the uncertainties of the measured temperatures of different components (i.e., RL and SM) are around 0.3–0.4 K, the averaged BT biases of 0.2 K among different coefficient sets (No.1–No. 3) are acceptable in general. Therefore, in practice, the coefficients labeled No. 2 are utilized for operational uses for FY-2F satellite.

**Table 2:** Comparison of calibration slopes for IR1–IR3 bands from different SIRC coefficients of FY-2F satellite with some typical cases in 2019

Time (MMDD_HHMM)	$T_{RL}$ (°C)	$T_{SM}$ (°C)	$CAL_{{\mathrm{slope}},IR1}$				$CAL_{{\mathrm{slope}},IR2}$				$CAL_{{\mathrm{slope}},IR3}$			
			No.1	No.2	No.3	$\sigma /{\mathrm{mean}}$	No.1	No.2	No.3	$\sigma /{\mathrm{mean}}$	No.1	No.2	No.3	$\sigma /{\mathrm{mean}}$
0101_0000	0.3	15.9	3.099	3.105	3.104	1.04‰	2.820	2.831	2.835	2.75‰	1.444	1.446	1.445	0.69‰
0201_0000	0.6	17.0	3.107	3.115	3.112	1.30‰	2.827	2.837	2.840	2.40‰	1.448	1.451	1.453	1.74‰
0301_0000	−4.2	15.9	3.030	3.035	3.034	0.87‰	2.749	2.757	2.763	2.55‰	1.417	1.419	1.419	0.81‰
0401_0000	−7.0	14.0	2.984	2.985	2.987	0.51‰	2.704	2.710	2.719	2.79‰	1.397	1.399	1.393	2.19‰
0501_0000	7.1	13.7	3.207	3.213	3.213	1.08‰	2.934	2.950	2.952	3.87‰	1.485	1.486	1.481	1.78‰
0601_0000	13.7	11.0	3.317	3.322	3.323	0.97‰	3.051	3.071	3.072	3.87‰	1.529	1.530	1.518	4.36‰
0701_0000	14.1	10.3	3.322	3.327	3.329	1.08‰	3.058	3.078	3.079	3.86‰	1.531	1.531	1.518	4.92‰
0801_0000	8.2	12.1	3.223	3.227	3.228	0.82‰	2.953	2.969	2.973	3.57‰	1.489	1.490	1.481	3.32‰
0901_0000	−6.6	13.1	2.987	2.987	2.990	0.58‰	2.709	2.716	2.725	2.95‰	1.397	1.398	1.391	2.71‰
1001_0000	−6.1	15.6	3.000	3.004	3.004	0.77‰	2.718	2.725	2.732	2.57‰	1.406	1.407	1.406	0.41‰
1101_0000	0.3	16.3	3.100	3.107	3.105	1.16‰	2.820	2.831	2.834	2.61‰	1.445	1.447	1.447	0.80‰
1203_0000	0.3	15.7	3.099	3.105	3.103	0.99‰	2.820	2.831	2.835	2.75‰	1.443	1.445	1.445	0.80‰
$\overline {\sigma /{\mathrm{mean}}}$, ${{{\mathrm{BT}}}}_{{\mathrm{bias}}}$			0.93‰, 0.05 K@290 K				3.04‰, 0.21 K@290 K				2.04‰, 0.17 K@250 K			

### Evaluation and implementation of SIRC

**Fig. 2:**
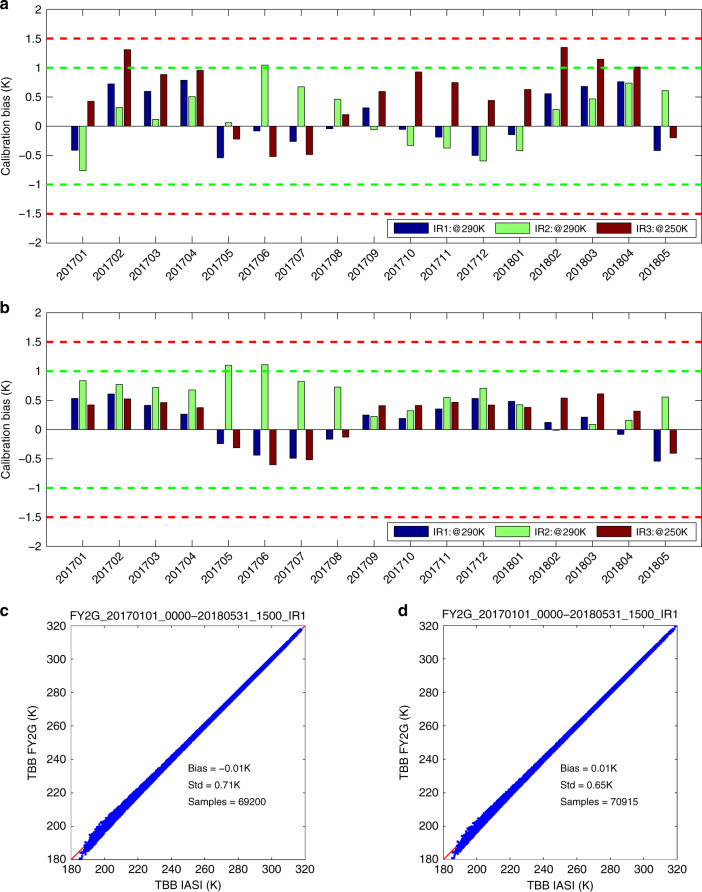
Comparison of calibration biases of FY-2G VISSR from both IBBC and SIRC methods for the period between January 2017 and May 2018. **a** Monthly biases of FY-2G VISSR IR1–IR3 bands with IBBC method; **b** Monthly biases of FY-2G VISSR IR1–IR3 bands with SIRC method; **c** Scatter plots of the collocated observations between FY-2G IR1 band and IASI with IBBC method; **d** Scatter plots of the collocated observations between FY-2G IR1 band and IASI with SIRC method

To evaluate the feasibility and the possible improvements of the proposed SIRC method, an approximate one-and-half-year period between January 2017 and May 2018 is selected, when the in-orbit radiometric calibration results of FY-2G satellite are reprocessed using SIRC for comparison with the operational ones from the previous IBBC. Specifically, the temperature measurements of RL and SM components together with the SIRC calibration coefficients listed in Table 1 for FY-2G satellite are utilized to generate recalibration results without its in-orbit internal BB views, which can produce different observed BT values from their original ones with IBBC results. Figures 2a and 2b provide the monthly averaged BT biases of FY-2G VISSR IR1–IR3 bands from IBBC and SIRC, respectively, which are less than 1 K for 90% cases with IBBC and for 96% cases with SIRC for all the results in the three bands. Meanwhile, the scattered plots of FY-2G VISSR IR1 band from the two methods are also shown in Figs. 2c and 2d, where the standard deviation (STD) of BT biases from IBBC (0.71 K) is slightly larger than that from SIRC (0.65 K) by analyzing around 70,000 samples while their mean values are reasonably identical (one is 0.01 K and the other is −0.01 K) with each other. It indicates that the vibration of the calibration biases of SIRC is decreased against that of IBBC thanks to the original internal BB view no longer utilized in SIRC, the uncertainty of which has no impact on the final calibration results at all. Moreover, in Figs. 2c and 2d, at the low value range around 180–200 K, the data point shows the larger uncertainty and deviating from the slope one, the root cause of which mainly comes from the nonlinear characteristics of the infrared photonic sensors utilized in FY-2G satellite. In fact, such a nonlinearity correction has not been implemented in FY-2 series due to insufficient in-lab calibration results more than 10 years ago, particularly for the low temperature range above.

**Fig. 3:**
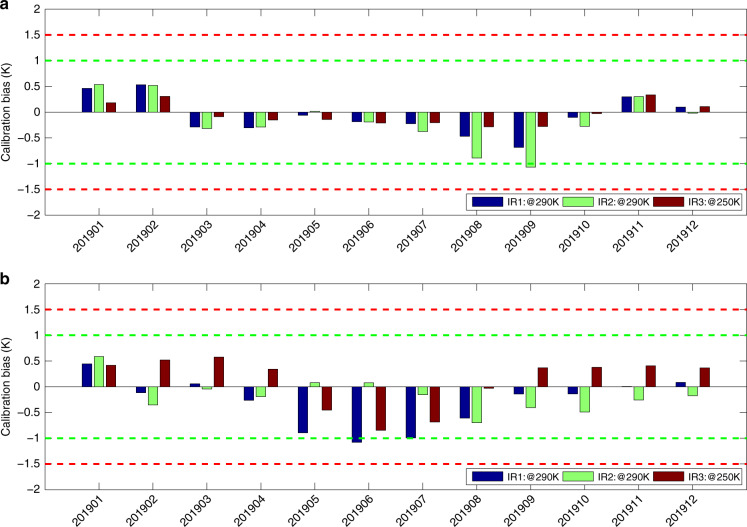
Monthly calibration biases of FY-2F and FY-2G VISSR with SIRC method for the period of 2019. **a** Reprocessed FY-2F results with SIRC method; **b** Operational FY-2G results with SIRC method. These calibration biases are intercalibrated with some reference hyperspectral sensors (i.e., IASI) under the framework of GSICS

Convinced on the above comparisons of FY-2G calibration results from the two methods, the SIRC one was operationally implemented for FY-2G and FY-2F satellites in January 2019 and January 2020, respectively, the calibration performance of which are being monitored online and supplemented by GSICS working groups in CMA to be less than 1 K for most IR bands (http://gsics.nsmc.org.cn/portal/en/fycv/xcal.html). In particular, for the period of the whole 2019, the evaluated monthly BT biases of the reprocessed for FY-2F (Notes: the reprocessing or called recalibration of FY-2F is identical with that of FY-2G from January 2017 to May 2018, with its own temperature measurements of the environmental fields as well as the SIRC calibration coefficients listed in Table 1) and the real-time processed for FY-2G, both of which are generated from SIRC method, are illustrated in Fig. 3. As expected, compared with the reference sensor (i.e., IASI), the monthly BT biases of the most IR bands (IR1–IR3) of FY-2F are lower than 0.5 K for 83% cases while those of FY-2G are less than 0.5 K for 72% cases. Such calibration performances achieved by both FY-2F and FY-2G in IR bands are the best among all the first-generation geostationary (spin stabilized for its attitude control) meteorological satellites in the world. As shown in Figs. 2a and 2b as well as Fig. 3, the monthly BT biases for both FY-2G and FY-2F satellites with either IBBC (belonging to SBRC) or SIBC method reveal significantly seasonal fluctuations. Although such fluctuations are not large enough with their amplitudes less than 1 K in general, the most likely cause lies in the seasonal temperature bias of each optical component (i.e., SM and RL), which is merely measured by a thermistor with the original purpose of monitoring its thermal variation under the in-orbit condition instead of calibration. However, due to the influence from the obliquity of the ecliptic during 1-year period, the uneven temperature field of each optical component caused by the different solar illumination cannot be represented by measurements from a single thermistor at all. Therefore, once the temperature values of individual component of a sensor are utilized for calibration, the measurement for its temperature field is required with multiple thermistors or platinum resistances for higher accuracy.

Currently, regarding the mid-infrared bands (IR4: 3.5–4.0 μm) of FY-2F and FY-2G satellites, their in-orbit radiometric calibration processings are not fulfilled directly with the proposed SIRC method due to lack of reliable enough intercalibrated results to generate the required coefficients for implementation. Specifically, when IASI or CrIS is used for reference, its spectral region does not cover the whole observation spectrum of IR4 band, which is a prominent limitation to assess the real calibrated biases of this band in an accurate way although some spectrum estimation technology can be applied for compensation^38^. Alternatively, an approximate method, which can be deduced from the proposed SIRC one, is thereafter implemented for the radiometric calibration of such a mid-infrared band by using the responsivity relationship established from the prelaunch testing results between one IR band (i.e., IR1) and this mid-infrared one (IR4), i.e.,

8 $$\ln \left( {CAL_{{\mathrm{slope}},IR4} - l_4} \right) = l_{14}^1 \cdot \ln \left( {CAL_{{\mathrm{slope}},IR1} - l_1} \right) + l_{14}^0$$

where the four constants (i.e., $l_{14}^1$, $l_{14}^0$, $l_1$, and $l_4$) are to be determined in different ways, the main results of which as provided in both Table S2 and Fig. S4. The more detailed descriptions about Eq. (8) can be found in Supplementary Note 8.

## Discussion

Based on some abnormal occurrence (i.e., invalid function of in-orbit internal BB view for FY-2G) as well as the more accurate requirement from some specialized observing situations (i.e., regional scanning mode for FY-2F), instead of the traditional SBRC one with BB source, the SIRC method independent of any onboard BB source for an IR sensor is originally proposed to be implemented for FY-2G and FY-2F in January 2019 and January 2020, respectively. Although the preliminary intention of the proposed SIRC is to resolve certain technical challenges encountered in practice, it becomes a universally satisfied methodology of in-orbit radiometric calibration for an IR photonic sensor with a closely background-limited capability, such performances of which have been widely achieved by most onboard payloads.

Specifically, a generally used equation for RC, describing the quantitative relationship between the calibration slope (namely radiometric responsivity) and the quantized background radiation in photons, is independently established for both two fundamental types (PC and PV) of HgCdTe photonic detectors in application of IR observation from space. Meanwhile, the SIRC can provide remarkable advantages in two aspects: one is low complexity to simplify both the design and the manufacture of onboard calibration components, where the traditional BB sources and their relevant assemblies are no longer required; the other is high accuracy to decrease the costs of traceability for measured radiation, where the uncertainties from BB sources are excluded. For example, an available BB with its emissivity between 0.990 and 0.995, the additionally introduced uncertainty in SBRC is around 0.2–0.5 K for the typical 10–11 μm band at the referenced 290 K. In practice, to achieve an acceptable calibration accuracy of less than 0.5 K, the uncertainty of temperature measurement is no more than 0.2 K. Meanwhile, since the frequency of onboard temperature measurements is usually at the order of 10^0^–10^1^ s, a realistic SIRC can be implemented in a temporal span of 5 m or less, which is well qualified for most infrared sensors onboard GEO or LEO platforms, using the average temperature measurements within the corresponding time intervals.

As recommended, the required calibration coefficients in SIRC can be obtained by solving a group of super-determined equations with a set of well-calibrated results (i.e., usually intercalibrated with some references, IASI or CrIS) for each band. To estimate more accurately, a 1-year period for the intercalibrated results is selected to cover all the possible background situations while the in-lab well-calibrated ones are doubtlessly feasible if such a similar coverage of background radiation can be satisfied, which means these coefficients can be determined before launch in theory. Moreover, due to lack of enough intercalibrated results to generate the required calibration coefficients, an approximate calibrating method for mid-infrared band is also deduced based on the proposed SIRC for implementation. In addition, the quadratic item for the traditional SBRC, which is usually determined before launch, can be also implemented to correct the possible nonlinear responsivity of an IR sensor with SIRC method.

Looking ahead, the SIRC will help a future IR micro-satellite constellation, which is hardly equipped with any BB source for calibration due to its limited capabilities in volume, mass, and energy. At the same time, all the IR sensors with such a SIRC method can be directly SI-traceable by using the measured temperature information of components, which contribute the background radiation in an accurate enough way (i.e., platinum transducer) under the in-orbit conditions. In particular, the SIRC is also suitable for those IR sensors, which do not strictly belong to (i.e., closely approached) BLIP ones but their dominated noise comes from photoeffects. Nevertheless, the more comprehensive in-lab tests prior to launch are in need to generate the required calibration coefficients of SIRC method for the convenience of the following utilization in space.

Since the early of 2019, the operational implementation of SIRC in both FY-2G and FY-2F satellites guarantees a long-term stable application of Chinese geostationary meteorological satellites for the global observation system under the framework of WMO, and further supports the meteorological services from FY-2 satellites for “B&R” countries. Furthermore, the SIRC method is also expected to recalibrate the measurements from all the FY-2 satellites to establish a 20-year-period traceable dataset for climate applications.

## Materials and methods

### Intercalibration between two IR sensors

Intercalibration or called cross-calibration aims to modify or correct the poorly calibrated results of the monitored (MON) sensor into the better ones according to the measurements from a reference (REF) sensor, where an ordinary simultaneous nadir observation mechanism is adopted to collocate the paired measurements from different sensors in temporal, spatial, and spectral aspects. Recommended by GSICS, some well-behaved hyperspectral IR sensors (i.e., AIRS, IASI, and CrIS) are regarded as REF sensors to calibrate others while the BT biases of the latter are extracted^36–38^. Particularly, in this article, we use these monitored BT biases of FY-2 VISSR in different IR bands to correct their original calibration slopes from IBBC method to the updated ones (namely intercalibrated results above), which implies that the BT biases are assumed to be mainly caused by the incorrect calibrated responsivities. This is the basic start point and its solution when applying intercalibration. More processing steps of intercalibration itself are not discussed here in detail.

### Calibration coefficients generation with super-determined linear equations

Equation (7) is a typical set of algebraic equations and provides a feasible way to solve or generate the required calibration coefficients for the proposed SIRC method. To cover the background radiation incident to a MCT detector as much as possible, the number (*N*_*s*_) of available equations from the intercalibrated results is practically larger than the number (*N*_*c*_ + 1) of unsolved known or coefficients, which makes these equations failed to be a unique solution in mathematics. In general, two classical methods, i.e., inversion and decomposition can be used, where the Gauss-Jordan elimination way for inverting a matrix is efficient enough among the inversion ones and some decomposition ways (i.e., low triangular and upper triangular, LU decomposition, and singular value decomposition SVD) are also adopted. Particularly, in many cases where Gaussian elimination and LU decomposition fail to give satisfactory results, SVD will diagnose precisely what the problem is and act as a reasonable choice for solving most linear least-squares problems as we encounter here. Thus, in our article, the SVD method is utilized to generate the calibration coefficients of SIRC, the more detailed discussions of which will not be done here.

## Supplementary information

Supplementary Notes

## Data Availability

FY-2F/G VISSR L1C data used in this work are publicly available at https://data.nsmc.org.cn. Other data supporting the findings of this study are available from the authors on reasonable request.
