# Supplementary material for: High-accuracy source-independent radiometric calibration with low complexity for infrared photonic sensors
Source: Light Sci Appl. 2021 Aug 9;10:163. doi: 10.1038/s41377-021-00597-4 (PMC8349907; doi:10.1038/s41377-021-00597-4)
Supplement: Supplementary file 1 — Supplementary Notes [file 41377_2021_597_MOESM1_ESM.docx]

**Supplementary Information for**

**High-accuracy source-independent radiometric calibration with low complexity for infrared photonic sensors**

Qiang Guo^1^*, Fuchun Chen^2^, Xiangyang Li^3^, Boyang Chen^1^, Xin Wang^1^, Guilin Chen^2^, and Caiying Wei^1^

*^1^ National Satellite Meteorological Center, China Meteorological Administration, Beijing 100081, China*

*^2^ Key Laboratory of Infrared System Detection & Imaging Technology, Shanghai Institute of Technical Physics, Chinese Academy of Sciences, Shanghai 200083, China*

*^3^ Key Laboratory of Infrared Imaging Materials & Detectors, Shanghai Institute of Technical Physics, Chinese Academy of Sciences, Shanghai 200083, China*

* Corresponding author. Email: [guoqiang@cma.gov.cn](mailto:guoqiang@cma.gov.cn)

**Supplementary Note 1: Abnormal situation of in-orbit calibration component of FY-2G**

The G satellite of Fengyun-2 series (FY-2G) was launched in December 31, 2014, in which an internal-blackbody (IBB) was adopted for its in-orbit radiometric calibration through a combination of both IBB-view and space-view operations^19^. A novel IBB calibration (IBBC) method was proposed in 2012 and thereafter applied in FY-2G satellite to achieve the calibration biases of less than 1K for all IR bands with some modifications of their spectral response functions^40^. Unfortunately, in June 2018, the IBB-view function of FY-2G was invalid caused by some unpredictably damaged components. Figure S1 shows the comparison of between the normal and the abnormal IBB-view results in IR1-IR4 bands of FY-2G satellite during the period from June to July 2018. Specifically, the plots of IR1-IR4 bands measured in 5 June were normal shown in Figure S1a, while those of both Figure S1b in June 25 and Figure S1c in July 1 behaved some abnormal features (the curves were almost constants) from the partially worse to the finally invalid for all. Due to lack of the necessary hourly IBB-views, as a temporary alternative, the IR measurements from FY-2G satellite were intercalibrated with IASI to generate the calibration coefficients for uses of every half a month from September to December, 2018. This situation promotes us to develop an original SIRC method instead of the traditional SBRC one and first apply it in FY-2G satellite in January 2019.

**Supplementary Note 2: Brief introduction of the regional scanning mode (RSM) for FY-2 satellite**

To achieve a more frequent observation upon some focused areas which suffer severe disasters (i.e. tropical cyclone and storm), the regional scanning mode (RSM) is designed and mainly implemented by the F satellite of this series (namely FY-2F) which is located in 112º E since November 2012. Usually, a 6-min time-interval is adopted for RSM of FY-2F which is 5 times faster than its ordinary full-disc observation for an interested region as given by a blue dotted area in Figure S2a, the coverage of which varies with time due to the non-zero orbit inclination angle of the satellite (i.e. FY-2F)^46^. The real examples are shown in both Figure S2b and Figure S2c, where the locations and their included angles relative to horizontal direction are slightly different. Meanwhile, the real scanning number during a 6-min period of FY-2F is generally less than 450, which occupies around 4.5 minutes and the rest 1.5 minutes are reserved for the reversed scanning as well as some necessary commands uploaded from ground to spacecraft. Therefore, the coverage width in North-South direction of a 6-min RSM is around 2500 km in general. Unfortunately, when a RSM is implemented, the IBB-view is forbidden due to the inevitable radiation intrusion from Earth scene. This is the main cause why such an IBBC operation cannot be done during the RSM of FY-2 satellite.

**Supplementary Note 3: Detective performances of Chinese IR sensors onboard GEO meteorological satellites**

A BLIP system is recommended for application in space^47^. Here, we merely provide the basic detective performances of some typical IR sensors onboard Chinese GEO meteorological satellites, where one type of simple detector unit with a single or a few PC and PV HgCdTe detector(s) is utilized. For the 1^st^ generation GEO meteorological satellite (FY-2), its main payload is VISSR with four IR bands covering the spectral region between 3.5 and 12.5 micrometers, where one PC detector is adopted for each band as listed in Table S1. In general, the real detectivities of three IR bands with a relatively longer wavelength, i.e. 6.3~7.6 μm, 10.3~11.3 μm and 11.5~12.5 μm approach (strictly less than) the ideal background-limited ones by around 10~20%^48^, the well-behaved performance of which implies that, the detectors in the three bands can be at least regarded as BLIP ones. As for the mid-infrared (3.5~4.0 μm) band, although its detectivity is not much closed to its background-limited one, the background radiation is still the dominated contributor to its detective performance since the two values remain in the same order with an acceptable relative departure less than 60~70%. For the 2^nd^ generation GEO meteorological satellite (Fengyun-4, FY-4), there are two IR sensors, i.e. Advanced Geostationary Radiation Imager (AGRI) and Geostationary Interferometric Infrared Sounder (GIIRS), the main spectral region and detector type information of which are also given in Table S1. Compared with FY-2 satellites, the PV HgCdTe detectors with a higher detective capability are used in both AGRI and GIIRS of FY-4A satellite instead of the traditional PC ones. On the whole, except for some longer wavelength bands (i.e. more than 13 μm), the real detectivities of all IR bands of both AGRI and GIIRS are good enough to be close to their ideal background-limited ones^49,50^. In fact, due to some big challenges in manufacture HgCdTe detectors in the spectral region beyond 13 μm, most applicable detectors in these bands approach by around 1/4~1/3 of their optimal background-limited performances, as shown in Table S1. At the same time, for the VISSR and AGRI, the background temperature for testing situation is the ordinary room temperature (around 298 K), while that of GIIRS is at a relatively lower one about 265 K thanks to its required cold aft-optics for a Fourier transfer sounder (FTS). Nevertheless, at least for Chinese sensors, the real detective performances of most IR bands are generally satisfied with requirements of a BLIP system.

**Supplementary Note 4: Relationships among different characteristics of a IR sensor**

Here, we will firstly review some figures of merit for a general photon detector in IR band, i.e.

**■ Noise equivalent power**

A convenient means to describe the output or observed noise of a photon detector is the conception of noise equivalent power ($NEP$), the radiant flux necessary to give an output signal equal to the detector noise, and can be written by either of the two different forms, namely

${NEP}_{PC}=\frac{P}{{v_{s}}/{v_{n}}}$ (s1-1)

${NEP}_{PV}=\frac{P}{{i_{s}}/{i_{n}}}$ (s1-2)

where $v_{s}/i_{s}$ is the root mean square (RMS) value of the photon detector’s output voltage/current when the incident radiant flux ($P$) with the unit of watt (W) is received, and $v_{n}/i_{n}$ is the RMS value of the noise voltage/current at the output of the detector. Here, Eqs. (s1-1) and (s1-2) are generally aimed for the PC and the PV detectors, respectively. Assuming that the irradiance ($H$) is mainly from an observed target whose spectral radiance can be given by $I_{obs}$ with unit of $W\cdot m^{-2}\cdot\mathrm{sr}^{-1}$ and the solid angle ($\Omega_{IFOV}$) subtended by the instant field-of-view (IFOV) of a detector whose area is given by $A_{d}$ with unit of $m^{2}$, the incident radiant flux ($P$) can be given by

${P=H\cdot A_{d}=I}_{obs}\cdot\Omega_{IFOV}\cdot A_{d}$ (s2)

**■ Detectivity**

When different detectors are compared in terms of their detecting capabilities, that is, the minimum detectable radiant flux, the best detector is the one with the lowest $\text{NEP}$. Hence, the detectivity ($\text{D}$) can be given by

$\text{D}=\frac{1}{\text{NEP}}$ (s3)

where the unit of $\text{D}$ is $W^{-1}$. It is found that the performance of $\text{D}$ is intrinsically influenced by at least six conditions of measurement, i.e. the wavelength of the incident radiant, the temperature of the detector, the chopping frequency, the bias current applied to the detector, the area of the detector and the bandwidth of the circuit used to measure detector noise. Moreover, extensive theoretical and experimental studies have shown that it is reasonable to assume that detectivity varies inversely as the square root of the detector’s area ($A_{d}$) as well as the electrical bandwidth ($\Delta f$). Therefore, the quantity $D^{*}$, the detectivity referred to an electrical bandwidth of 1 Hz and a detector area of 1 cm^2^, can be described as following

$D^{*}=\frac{\sqrt{A_{d}\cdot\Delta f}}{NEP}$ (s4)

Apparently, $D^{*}$ is a normalized detectivity to compare the performance of detectors with different areas when used in circuits with different bandwidths.

**■ Basic relationship between different figures of merit**

Substituting Eqs. (s1-s2) into Eq. (s4), we can draw

${v_{s}=\left( \Omega_{IFOV}\cdot\sqrt{\frac{A_{d}}{\Delta f}}\cdot D^{*}\cdot v_{n} \right)\cdot I}_{obs}$ (s5-1)

${i_{s}=\left( \Omega_{IFOV}\cdot\sqrt{\frac{A_{d}}{\Delta f}}\cdot D^{*}\cdot i_{n} \right)\cdot I}_{obs}$ (s5-2)

where Eqs. (s5-1) and (s5-2) are suitable for the PC and the PV photon detector systems respectively.

**Supplementary Note 5: Derivation of responsivity for a PC-type BLIP sensor**

According to the definition of $NEP$ in Eqs. (s1), the minimum detectable radiant flux is absolutely dominated by the total output noise level ($v_{n}$), which usually consists of three main components for a PC detector, i.e. the thermal noise, the $1/f$ noise, and the generation and recombination noise^51^. For some real space uses, on the one hand, the photon detector is ordinarily operated at a sufficiently low temperature (around 80~90 K or less) to make the RMS value of the thermal noise to be neglectable among the total one. On the other hand, for a pulse-type system (i.e. FY-2/VISSR), the contribution of its $1/f$ noise can be ignored due to the employed relatively higher chopping or sampling frequency (more than 10^6^ Hz); for a staring-type one where a focal-plane-array sensor with multiple detectors is generally adopted, the influence of its $1/f$ noise can also be decreased to a great extent by adjusting the offset configuration (current or voltage) through the device.

Currently, most onboard passive infrared systems are well designed to be limited by the generation and recombination noise ($v_{g-r}$) in their detecting elements, where the generation of carriers is dominantly due to photon excitation. As a result, their radiation responsivity variations are determined in the ideal case by the equivalent temperature of the background against which a target appears. So, for a BLIP one, $v_{n}$ in Eq. (s5-1) can be given by

$v_{n}\cong v_{g-r}$ (s6)

Due to fluctuations in the rate at which charge carriers are generated and recombined, the power spectrum of g-r noise is flat and approximately equal to the inverse of the free carrier lifetime. Particularly, for a photoconductor, its g-r noise within $\Delta f$ bandwidth is^52^

$v_{g-r}=2\cdot V_{a}\cdot\left[ \frac{p\cdot\tau\cdot\Delta f}{n\cdot\left( n+p \right)\cdot A_{d}\cdot x_{t}} \right]^{1/2}$ (s7)

where $V_{a}$ represents the detector applied (or called bias) voltage, $x_{t}$ is the thickness of the detector, $\tau$ is the effective photoconductive lifetime, and $p$ and $n$ represent the minority carrier density and the free electron density respectively. Meanwhile, for a BLIP PC detector, its normalized detectivity ($D^{*}$) is theoretically satisfied with^52^

$D^{*}=\frac{\eta_{PC}}{2\cdot E_{g}}\cdot\left[ \frac{\tau}{x_{t}}\cdot\frac{n+p}{n\cdot p} \right]^{1/2}$ (s8)

where $\eta_{PC}$ represents the quantum efficiency of a PC photon detector and $E_{g}$ is the band-gap energy. With Eqs. (s6-s8), Eq. (s5-1) can be rewritten as

${I_{obs}=\frac{x_{t}\cdot E_{g}}{\eta_{PC}\cdot\tau\cdot\Omega_{IFOV}\cdot V_{a}}\cdot n\cdot v}_{s}$ (s9)

Under the condition of excess carrier generation by a background photon flux ($\Phi_{B}$), the electron density ($n$) can be expressed by^52^

$n=n_{0}+\frac{\eta_{PC}\cdot\tau}{x_{t}}\cdot\Phi_{B}$ (s10)

where $n_{0}$ is the thermal equilibrium electron carrier concentration of the material (i.e. MCT), which is determined by the operating temperature of detectors. Substituting Eq. (s10) into Eq. (s9), then

${I_{obs}=\left( \xi_{PC}^{0}+\xi_{PC}^{1}\cdot\Phi_{B} \right)\cdot v}_{s}$ (s11)

where

$\xi_{PC}^{0}=\frac{{n_{0}\cdot x_{t}\cdot E}_{g}}{\eta_{PC}\cdot\tau\cdot\Omega_{IFOV}\cdot V_{a}}>0$ (s12-1)

$\xi_{PC}^{1}=\frac{E_{g}}{\Omega_{IFOV}\cdot V_{a}}>0$ (s12-2)

In Eqs. (s11-s12), for a given PC-type BLIP system in space, since its operating temperature and chopping frequency are generally fixed and its quantum efficiency ($\eta_{PC}$) is theoretically unrelated with background radiation ($\Phi_{B}$), the parameters $\xi_{PC}^{0}$ and $\xi_{PC}^{1}$ are reasonably regarded as the positive constants to be determined.

**Supplementary Note 6: Derivation of responsivity for a PV-type BLIP sensor**

For a given PV type BLIP system, its normalized detectivity ($D^{*}$) and the RMS value of its noise current ($i_{n}$) can be given by^41(p203-204 & p58)^

$D^{*}=\eta_{PV}\frac{\lambda\cdot q}{hc}\left( 2q^{2}\cdot\eta_{PV}{\cdot\Phi}_{B} \right)^{-1/2}$ (s13-1)

$i_{n}=\sqrt{A_{d}\cdot\Delta f}\cdot\left( 2q^{2}\cdot\eta_{PV}{\cdot\Phi}_{B} \right)^{1/2}$ (s13-2)

where $h$ is the Planck constant, $c$ is the velocity of light, $q$ is the charge of one electron, $\lambda$ is the wavelength, $\eta_{PV}$ is the quantum efficiency of a PV detector, and the definitions of the other parameters (i.e. $A_{d}$, $\Delta f$ and $\Phi_{B}$) can be referred to the above statements. Substituting Eqs. (s13) into Eq. (s5-2), we can draw

${i_{s}=\left( \eta_{PV}\cdot\frac{\lambda\cdot q\cdot\Omega_{IFOV}\cdot A_{d}}{hc} \right)\cdot I}_{obs}$ (s14)

According to the operating mechanism of a PV detector system, three regions contribute to its quantum efficiency ($\eta_{PV}$): two neutral regions of different types (p and n) of conductivity and the spatial charge region. Thus^41(p201)^:

$\eta_{PV}=\eta_{n}+\eta_{DR}+\eta_{p}$ (s15)

where $\eta_{n}$ and $\eta_{p}$ are the quantum efficiencies from both the n-type and p-type neutral regions respectively and $\eta_{DR}$ is the quantum efficiency from the depletion region (DR). In practice, the main contribution of the total $\eta_{PV}$ is from the two neutral regions ($\eta_{n}+\eta_{p}$), both of which usually remain stable. Therefore, Eq. (s15) can be simplified to be

$\eta_{PV}\cong\psi_{0}+\psi_{1}\cdot e^{-\bar{\alpha}\cdot w}$ (s16)

where $\bar{\alpha}$ is the mean absorption coefficient of a PV detector within its observation spectral region, $w$ is the width of the DR for spatial charge, $\psi_{0}$ and $\psi_{1}$ are two positive constants. In reality, considering that $\bar{\alpha}$ is at the order of 10^5^ m^-1^ or less and $w$ is less than 1 μm, $\left\| \bar{\alpha}\cdot w \right\|\ll1$ is always satisfied and Eq. (s16) can be rewritten as

$\eta_{PV}\cong\psi_{0}+\psi_{1}\cdot\left( 1-\bar{\alpha}\cdot w \right)$ (s17)

The width of the DR for spatial charge at the thermal equilibrium state can be given by^41(p207)^:

$w_{eq}=\sqrt{\frac{2\epsilon}{q}\cdot\frac{N_{a}+N_{d}}{N_{a}N_{d}}\cdot\left( V_{bi}-V_{a} \right)}$ (s18-1)

$V_{bi}=\frac{k\cdot T_{d}}{q}\cdot\ln\left( \frac{N_{a}N_{d}}{n_{0}^{2}} \right)$ (s18-2)

where $V_{bi}$ is the built-in voltage, $\epsilon$ is the dielectric constant of MCT material, $N_{a}$ and $N_{d}$ are the acceptor and donor concentrations respectively, $T_{d}$ is the working temperature of detector, and $k$ is the Boltzmann constant. Using Eqs. (s18), we can draw

$w_{eq}\triangleq w_{0}\cdot\sqrt{\left[ \ln\left( \frac{N_{a}N_{d}}{n_{0}^{2}} \right)-V_{a}' \right]}\cong w_{0}\cdot\sqrt{\left( \frac{N_{a}N_{d}}{n_{0}^{2}}-1-V_{a}' \right)}$ (s19-1)

$w_{0}=\sqrt{\frac{2\epsilon}{q}\cdot\frac{N_{a}+N_{d}}{N_{a}N_{d}}\cdot\frac{k\cdot T_{d}}{q}}$ (s19-2)

$V_{a}^{'}=\frac{q}{k\cdot T_{d}}\cdot V_{a}$ (s19-3)

In real applications, to increase the performance (i.e. $D^{*}$) of a PV-type BLIP system as much as possible, a feasible way is to adjust the applied voltage ($V_{a}$) in order to make it sure that $w_{eq}$ approaches zero. For Eq. (s19-1), it means

$\left( \frac{N_{a}N_{d}}{n_{0}^{2}}-1-V_{a}' \right)\approx0$ (s20)

Apparently, when a PV-type BLIP system is faced to the incident background radiation $\Phi_{B}$, using Eqs. (s18), the width of the DR for spatial charge ($w_{\Phi_{B}}$) and the corresponding built-in voltage ($V_{bi,\Phi_{B}}$) can be given by

$V_{bi,\Phi_{B}}=\frac{k\cdot T_{d}}{q}\cdot\ln\left( \frac{N_{a}N_{d}+\left( q\cdot\Phi_{B} \right)^{2}}{n_{0}^{2}} \right)$ (s21-1)

$w_{\Phi_{B}}=w_{0}\cdot\sqrt{\ln\left( \frac{N_{a}N_{d}+\left( q\cdot\Phi_{B} \right)^{2}}{n_{0}^{2}} \right)-V_{a}^{'}}\cong w_{0}\cdot\sqrt{\left( \frac{q\cdot\Phi_{B}}{n_{0}} \right)^{2}+\left( \frac{N_{a}N_{d}}{n_{0}^{2}}-1-V_{a}^{'} \right)}$ (s21-2)

Substituting Eq. (s20) into Eq. (s21-2), then

$w_{\Phi_{B}}\cong\frac{w_{0}\cdot q}{n_{0}}\cdot\Phi_{B}$ (s22)

Assuming the load impedance of a PV-type detector to be invariable (${IMP}_{L}$), its output voltage $v_{s}$ can be given by

$v_{s}={IMP}_{L}\cdot i_{s}$ (s23)

Substituting Eqs. (s17), (s22) and (s23) into Eq. (s14), we can draw

$I_{obs}=\left( \xi_{PV}^{0}+\xi_{PV}^{1}\cdot\Phi_{B} \right)^{-1}\cdot v_{s}$ (s24)

where

$\xi_{PV}^{0}=\frac{\lambda\cdot q\cdot\Omega_{IFOV}\cdot A_{d}}{hc\cdot{IMP}_{L}}\cdot\left( \psi_{0}+\psi_{1} \right)>0$ (s25-1)

$\xi_{PV}^{1}=-\frac{\lambda{\cdot q}^{2}\cdot\Omega_{IFOV}\cdot A_{d}\cdot\bar{\alpha}\cdot w_{0}}{hc\cdot{IMP}_{L}\cdot n_{0}}\cdot\psi_{1}<0$ (s25-2)

Similarly, in Eqs. (s24-s25), for a given PV-type BLIP system in space, since its operating temperature and other environmental situations are generally stable, the parameters $\xi_{PV}^{0}$ and $\xi_{PV}^{1}$ are doubtlessly regarded as the constants to be determined, where the former is positive and the latter is negative.

**Supplementary Note 7: Basic observation mechanism of FY-2 VISSR**

In theory, a sensor, e.g. FY-2 VISSR in thermal infrared band, will gather all the radiation within the incident direction emitted from both the interested target and itself, which is usually called background radiation and determines the responsivity characteristics of a sensor particularly for a BLIP system. Figure S3 illustrates the optical path sketch of FY-2 VISSR^19^, where the positional relationship among the main optical components, e.g. the primary mirror (PM), the secondary mirror (SM), the folding mirror (FM), the calibration mirror (CM), the relay lens(RL) and the internal BB is clearly illustrated. For the normal (space- or Earth- view) observation, the target’s radiation is gathered by VISSR in form of parallel beam and then converged to the detectors by three-time reflection by PM, SM and FM in order, as well as one penetration of RL. Theoretically, the background radiation of VISSR contributes from PM, SM, FM and RL in total, where the reflectivities of the three mirrors are usually larger than 0.98 whilst the transmittance or emissivity of RL is also more than 0.97 for all the four infrared bands. Therefore, for FY-2 VISSR, the main contributor of its background radiation comes from RL. However, due to the relatively larger field angle of SM to gather more radiation, parts of frame structure of SM with a non-ignorable emissive feature (around 0.8~0.9) can be viewed directly by each infrared detector, which implies that the self-radiation of SM is also included in modeling the total background one. It should be emphasized that, nevertheless, what components should be considered for background radiation cannot be fixed for different sensors and can be determined according to its optical and mechanical structures.

**Supplementary Note 8: In-orbit approximate calibration method for mid-infrared (3.5~4.0 μm) of FY-2 satellites**

Regarding the $r^{th}$ band of a BLIP sensor, its calibration slope (namely radiometric responsivity) by using Eq.(6) can be given by

${CAL}_{slope,r}=\left\{ \left( \xi_{X}^{0} \right)_{r}+\left( \xi_{X}^{1} \right)_{r}\cdot\Phi_{B,r} \right\}^{m_{r}}$ (s26)

Specifically, for FY-2 VISSR, since all the adopted IR detectors belong to the PC-type, $m_{r}$ in Eq.(s26) equals unit. Thus, by applying logarithm on both sides of Eq.(s26), it can be rewritten as

$\ln\left[ {CAL}_{slope,r}-\left( \xi_{PC}^{0} \right)_{r} \right]=\ln\left[ \left( \xi_{PC}^{1} \right)_{r} \right]+\ln\left( \Phi_{B,r} \right)$ (s27)

When $r$ is set to be IR1 and IR4 respectively, it is generally acceptable to use the radiances at the averaged wavelengths $\bar{\lambda}_{IR1}$ and $\bar{\lambda}_{IR4}$ based on their SRFs instead of those weighted radiances at different wavelengths within the regions covered by their SRFs. Meanwhile, since the contribution of RL to the total background radiation is significant larger than that of SM particularly for FY-2F as indicated in Table 1, it is reasonable to simply Eq.(s27) to be

$\Phi_{B,IR1}=\sum_{i} \Phi_{B,IR1}^{i}\cong\sum_{i} \left[ \zeta_{i}\cdot Q\left( \bar{\lambda}_{IR1},T_{i} \right) \right]\approx\zeta_{RL}\cdot Q\left( \bar{\lambda}_{IR1},T_{RL} \right)$ (s28-1)

$\Phi_{B,IR4}=\sum_{i} \Phi_{B,IR4}^{i}\cong\sum_{i} \left[ \zeta_{i}\cdot Q\left( \bar{\lambda}_{IR4},T_{i} \right) \right]\approx\zeta_{RL}\cdot Q\left( \bar{\lambda}_{IR4},T_{RL} \right)$ (s28-2)

Applying logarithm on both sides of Eqs.(s28), then

$\ln\left( \Phi_{B,IR1} \right)\cong\ln\left( \zeta_{RL}\cdot\frac{2\pi c}{\bar{\lambda}_{IR1}^{4}}\cdot\frac{1}{e^{{hc}/{\bar{\lambda}_{IR1}kT_{RL}}}} \right)=\ln\left( \zeta_{RL}\cdot\frac{2\pi c}{\bar{\lambda}_{IR1}^{4}} \right)-\frac{hc}{\bar{\lambda}_{IR1}kT_{RL}}$ (s29-1)

$\ln\left( \Phi_{B,IR4} \right)\cong\ln\left( \zeta_{RL}\cdot\frac{2\pi c}{\bar{\lambda}_{IR4}^{4}}\cdot\frac{1}{e^{{hc}/{\bar{\lambda}_{IR4}kT_{RL}}}} \right)=\ln\left( \zeta_{RL}\cdot\frac{2\pi c}{\bar{\lambda}_{IR4}^{4}} \right)-\frac{hc}{\bar{\lambda}_{IR4}kT_{RL}}$ (s29-2)

Substituting Eqs.(s29) into Eq.(s27), it becomes

$\ln\left[ {CAL}_{slope,IR1}-\left( \xi_{PC}^{0} \right)_{IR1} \right]-\ln\left[ \left( \xi_{PC}^{1} \right)_{IR1}\cdot\zeta_{RL}\cdot\frac{2\pi c}{\bar{\lambda}_{IR1}^{4}} \right]=-\frac{hc}{\bar{\lambda}_{IR1}k}\cdot\frac{1}{T_{RL}}$ (s30-1)

$\ln\left[ {CAL}_{slope,IR4}-\left( \xi_{PC}^{0} \right)_{IR4} \right]-\ln\left[ \left( \xi_{PC}^{1} \right)_{IR4}\cdot\zeta_{RL}\cdot\frac{2\pi c}{\bar{\lambda}_{IR4}^{4}} \right]=-\frac{hc}{\bar{\lambda}_{IR4}k}\cdot\frac{1}{T_{RL}}$ (s30-2)

With Eqs.(s30), we can draw

$\ln\left( {CAL}_{slope,IR4}-l_{4} \right)=l_{14}^{1}\cdot\ln\left( {CAL}_{slope,IR1}-l_{1} \right)+l_{14}^{0}$ (s31)

where

$l_{14}^{1}=\frac{\bar{\lambda}_{IR1}}{\bar{\lambda}_{IR4}}$ (s32-1)

$l_{14}^{0}=\frac{\bar{\lambda}_{IR4}\cdot\ln\left[ \left( \xi_{PC}^{1} \right)_{IR4}\cdot\zeta_{RL}\cdot\frac{2\pi c}{\bar{\lambda}_{IR4}^{4}} \right]-\bar{\lambda}_{IR1}\cdot\ln\left[ \left( \xi_{PC}^{1} \right)_{IR1}\cdot\zeta_{RL}\cdot\frac{2\pi c}{\bar{\lambda}_{IR1}^{4}} \right]}{\bar{\lambda}_{IR4}}$ (s32-2)

${l_{1}=\left( \xi_{PC}^{0} \right)}_{IR1}$ (s32-3)

${l_{4}=\left( \xi_{PC}^{0} \right)}_{IR4}$ (s32-4)

Here, $l_{14}^{1}$ can be easily obtained from its definition, $l_{1}$ is the deduced coefficient of SIRC method, and the rest two constants ($l_{14}^{0}$ and $l_{4}$) can be estimated using the prelaunch testing results according to the established relationship given by Eq.(s31). The real values of the four constants for both FY-2F and FY-2G satellites are listed in Table S2. It is shown that the $l_{14}^{1}$ constants are merely related with spectral characteristics of sensor and nearly identical between FY-2F and FY-2G satellites, and three other ones are dominated by the responsive features of different detectors and reasonably differ with each other. Furthermore, the approximate responsivity (i.e. calibration slopes) relationships between IR1 and IR4 bands for two FY-2 satellites are illustrated in Figure S4, where six groups of the prelaunch testing results are selected for each sensor to determine $l_{14}^{0}$ and $l_{4}$, and two linear fitted curves are almost parallel and utilized to estimate the calibration slopes of the mid-infrared (IR4 band) of FY-2F and FY-2G, respectively.

**References**

1. F. Lu, *et al.* Image navigation for the FY2 geosynchronous meteorological satellite. *Journal of Atmospheric and Oceanic Technology* **25**, 1149-1165 (2008)
2. I. H. Swift. Performance of background-limited systems for space use. *Infrared Physics* **2**, 19-30 (1962)
3. B. Chen, *et al.* Infrared Imaging Detectors for Space Applications. (CSMP, Beijing, China, 2016)
4. X. Li, *et al.* How the smallest clairvoyance in meteorological satellite was tempered? -- The preparation of the infrared-detector chips of FY-4A multiple channel scanning radiation imager. *Chinese Journal of Nature* **40(2)**, 90-101 (2018)
5. J. Hua, *et al.* Review of Geostationary Interferometric Infrared Sounder, *Chinese Optics Letters* **16(11)**, 111203 (2018)
6. D. Long. Generation-recombination noise limited detectivities of impurity and intrinsic pc 8-14μm infrared detectors. *Infrared Physics* **7**, 121-128 (1967)
7. S. Borrello, *et al.* Photoconductive HgCdTe detector performance with background variations. *Infrared Physics* **17**, 121-125 (1977)

**
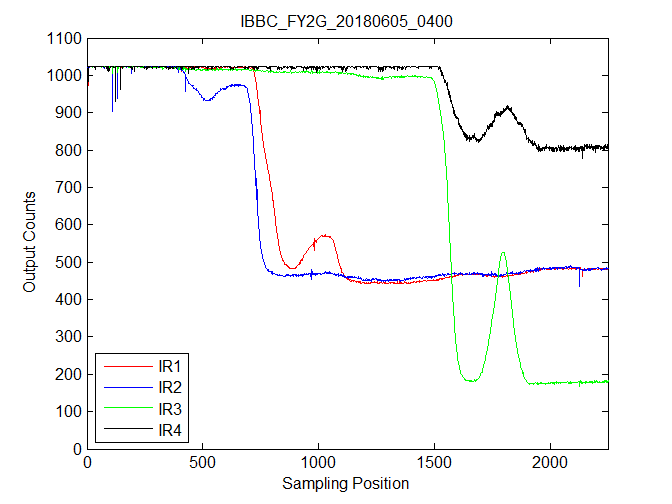

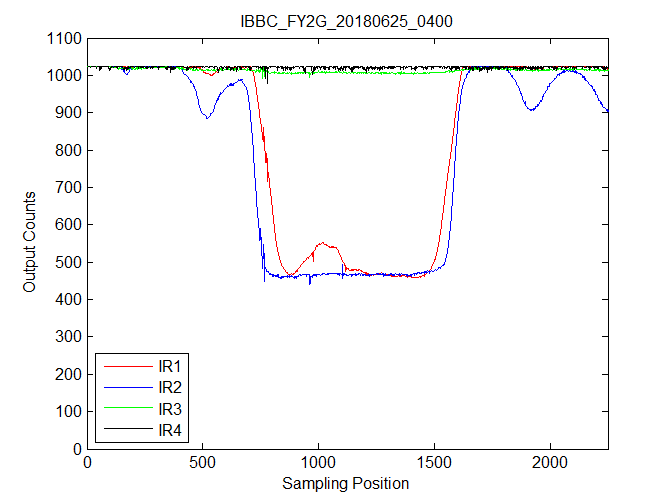

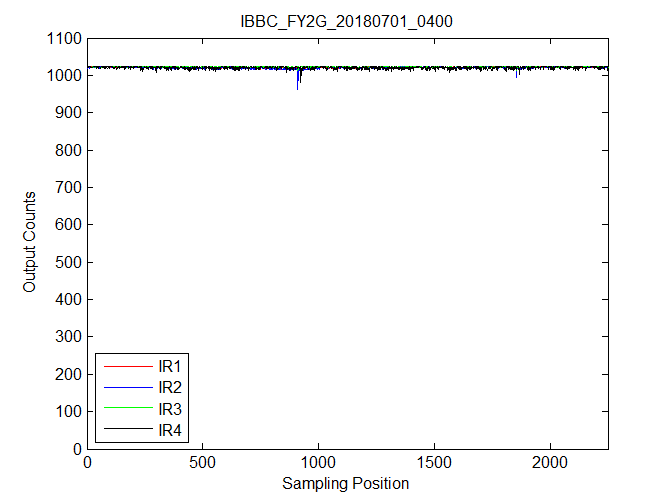
**

(a) (b) (c)

**Figure S1. Comparison of the viewed IBB results in all IR bands of FY-2G satellite under between the normal and the abnormal situations.** (a) IBB-views in 5 June 2018; (b) IBB-views in 25 June 2018; (c) IBB-views in 1 July 2018. The curves of IR1-IR4 bands shown in Figure S1a measured in June 5 2018 are normal, the minimal averaged values within a given-width (i.e. 5 or 7 samples) region of which represent the valid IBB measurements for each band, for example ~ 470 counts for both IR1 and IR2, ~180 counts for IR3 and ~800 counts for IR4. It should be pointed out that, these counts are generally reversed from its original ones to be easily as a matter of convenience for display. Similar curves are provided in Ref. #19. However, for Figure S1b in June 25 2018, the curves of IR3 and IR4 are almost constants (~1023, their revered ones are zeros) while partial samples at the locations beyond 1500 are abnormal comparative to those in Figure S1a. Unfortunately, for Figure S1c in July 1 2018, all the curves of the four IR bands approach 1023, which means no valid IBB-measurement is available.


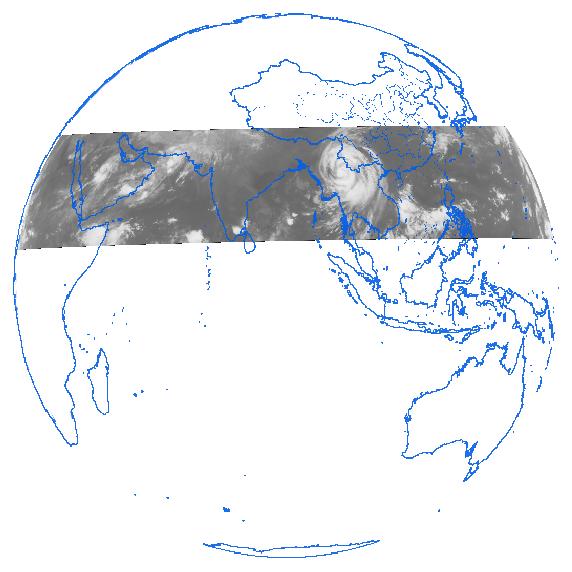

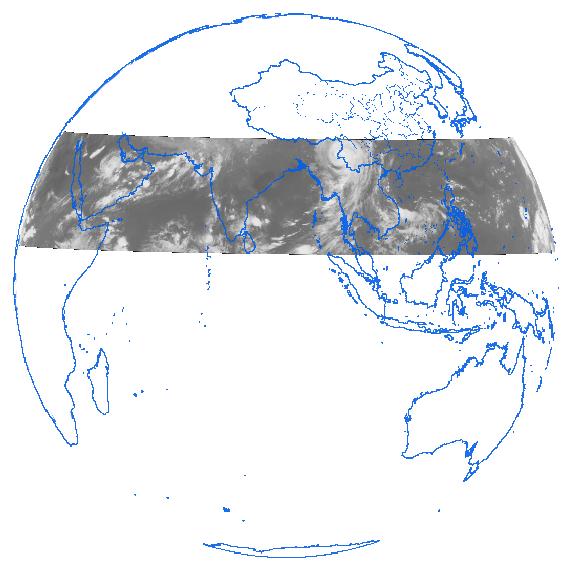


(a) (b) (c)

**Figure S2. Illustration of regional scanning mode (RSM) of FY-2F satellite.** (a)Viewable region illustration of Earth scene for RSM; (b) Example of FY-2F RSM in UTC0000 30 September 2019; (c) Example of FY-2F RSM in UTC1330 30 September 2019. In Figure S2a, the area of blue dash square represents the aimed one of a RSM observation, the typical imaginaries of which are shown in Figures S2b and S2c respectively with a slightly different coverages in both location and orientation. Specifically, in Figure S2a, the location of an observed region of RSM shifts in the North-South direction given by the blue two-directional arrow, and its orientation described by the included angle (δ) is also variable with time.

**Table S1. Comparison between the background-limited and the real-performed D* for some Chinese spaceborne IR sensors on GEO platforms**

| Satellite | Sensor | Band (μm) | Type of MCT Detector | Background Temperature (K) | Field of View (º) | Background-limited Value of D*  (cm.Hz^1/2^.W^-1^) | Real-performed of D*  (cm.Hz^1/2^.W^-1^) |
| --- | --- | --- | --- | --- | --- | --- | --- |
| FY-2  (F/G/H) | VISSR | 3.5~4.0 | PC | 298 | 60 | 5.7×10^11^ | 3.5×10^11^ |
|  |  | 6.3~7.6 | PC | 298 | 60 | 1.2×10^11^ | 1.1×10^11^ |
|  |  | 10.3~11.3 | PC | 298 | 60 | 1.4×10^11^ | 1.3×10^11^ |
|  |  | 11.5~12.5 | PC | 298 | 60 | 1.6×10^11^ | 1.3×10^11^ |
| FY-4A | AGRI  (imager) | 3.5~4.0 | PV | 298 | 30 | 1.5×10^12^ | 1.2×10^12^ |
|  |  | 5.8~6.7 | PC | 298 | 60 | 1.5×10^11^ | 1.2×10^11^ |
|  |  | 6.75~7.15 | PC | 298 | 60 | 2.0×10^11^ | 1.6×10^11^ |
|  |  | 8.3~8.8 | PC | 298 | 60 | 1.8×10^11^ | 1.8×10^11^ |
|  |  | 10.3~11.3 | PC | 298 | 60 | 1.4×10^11^ | 1.2×10^11^ |
|  |  | 13.0~13.6 | PC | 298 | 60 | 2.2×10^11^ | 7.0×10^10^ |
|  | GIIRS  (sounder) | 4.44~6.06 | PV | 265^(1)^ | 52 | 4.1×10^11^ | 4.0×10^11^ |
|  |  | 8.85~14.3 | PC | 265^(1)^ | 52 | 1.6×10^11^ | 5.0×10^10^ |

*Note*(1): Since GIIRS is a FTS sounder operated on a relative cold temperature (200K) for its aft-optics, its equivalent background temperature is estimated to be around 265K, much lower than the environment one (298K) for most imagers, i.e. VISSR and AGRI.

**Figure S3. Optical path sketch of FY-2 VISSR.** Traditionally, the internal blackbody view is adopted for in-orbit radiometric calibration by rotating the calibration mirror to make it to be observed by each infrared detector, where the different background radiations between the space-to-Earth and the internal blackbody views should be considered. However, since the detectivity characteristics of VISSR almost approach the ideal background-limited ones, the radiation contributions from PM, SM, FM and RL for a space-to-Earth view should be considered and analyzed in the relevant contents in Supplementary Note 7.

**Table S2. Main deduced constants for in-orbit approximate calibration method for mid-infrared (3.5~4.0 μm) of FY-2 satellites**

| **Satellite** | $\boldsymbol{l}_{\boldsymbol{14}}^{\boldsymbol{1}}$ | $\boldsymbol{l}_{\boldsymbol{14}}^{\boldsymbol{0}}$ | $\boldsymbol{l}_{\boldsymbol{1}}\left( \boldsymbol{\xi}_{\boldsymbol{IR}\boldsymbol{1}}^{\boldsymbol{0}} \right)$ | $\boldsymbol{l}_{\boldsymbol{4}}\left( \boldsymbol{\xi}_{\boldsymbol{IR}\boldsymbol{4}}^{\boldsymbol{0}} \right)$ |
| --- | --- | --- | --- | --- |
| FY-2F | 2.875398^*^ | -2.813060 | 1.974135^**^ | 0.358433 |
| FY-2G | 2.897215^*^ | -3.226095 | 1.561097^**^ | 0.295501 |

***Note***: (*) these constants can be obtained from their definitions. (**) these constants are adopted directly from Table 1. The other constants filled in gray columns are estimated based on Eqs.(s32) with in-lab testing results prior to launch.


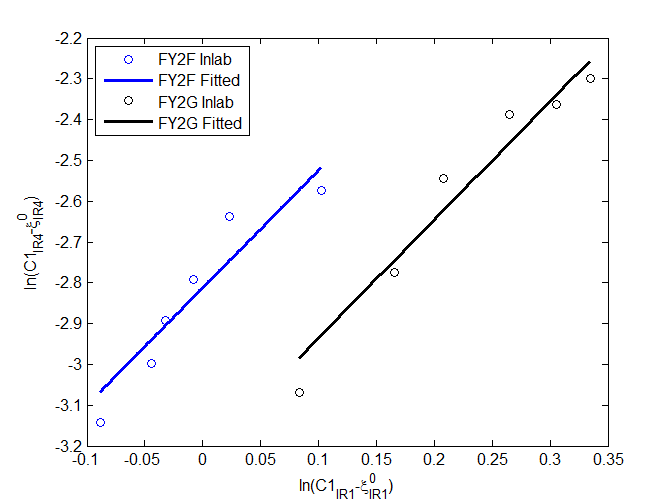


**Figure S4. Approximate responsivity relationship of different bands (i.e. IR1 vs. IR4) onboard the same FY-2 satellite.**
